# Heteroploid reticulate evolution and taxonomic status of an endemic species with bicentric geographical distribution

**DOI:** 10.1093/aobpla/plx002

**Published:** 2017-01-25

**Authors:** Kai Uwe Nierbauer, Juraj Paule, Georg Zizka

**Affiliations:** 1 Botany and Molecular Evolution, Senckenberg Research Institute and Natural History Museum Frankfurt, Senckenberganlage 25, D-60325 Frankfurt am Main, Germany; 2 Diversity, Evolution and Phylogeny of Higher Plants and Lichens, Institute for Ecology, Evolution and Diversity, Goethe University, Max-von-Laue-Str. 13, D-60438 Frankfurt am Main, Germany

**Keywords:** AFLP, *Campanula* sect. *Heterophylla*, cpDNA, flow cytometry, hybridization, ITS, polyploidy, *rpl*32-*trn*L

## Abstract

Reticulate evolution is considered to be among the main mechanisms of plant evolution, often leading to the establishment of new species. However, complex evolutionary scenarios result in a challenging definition of evolutionary and taxonomic units. In this study, we aimed to examine the evolutionary origin and revise the species status of *Campanula baumgartenii*, a rare endemic species from the polyploid complex *Campanula* section *Heterophylla.* Morphometry, flow cytometric ploidy estimation, amplified fragment length polymorphisms (AFLPs), as well as chloroplast and nuclear DNA sequence markers were used to assess the morphological and genetic differentiation among *C. baumgartenii*, *Campanula rotundifolia* and other closely related taxa. Tetra- and hexaploid *C. baumgartenii* is morphologically and molecularly (AFLP) differentiated from sympatric *C. rotundifolia.* Contrasting signals from nuclear (ITS) and chloroplast (*trn*L-*rpl*32) markers suggest a hybrid origin of *C. baumgartenii* with *C. rotundifolia* and a taxon related to the alpine *Campanula scheuchzeri* as ancestors. Additionally, hexaploid *C. baumgartenii* currently hybridizes with co-occurring tetraploid *C. rotundifolia* resulting in pentaploid hybrids, for which *C. baumgartenii* serves as both seed and pollen donor. Based on the molecular and morphological differentiation, we propose to keep *C. baumgartenii* as a separate species. This study exemplifies that detailed population genetic studies can provide a solid basis for taxonomic delimitation within *Campanula* section *Heterophylla* as well as for sound identification of conservation targets.

## Teaser

Campanula baumgartenii is an endemic species of the polyploid complex Campanula section Heterophylla with a peculiar bicentric distribution. C. baumgartenii often grows in sympatry with other morphologically similar Campanula species (C. rotundifolia). Hence its species status has been doubtful. Using a multimethodological approach we confirmed the species status of C. baumgartenii and uncovered complex patterns of genetic and ploidal variability. These were identified as resulting from dynamic and ongoing evolutionary processes including several hybridization and backcrossing events. This study also illustrates that detailed population genetic studies can provide a solid basis for sound identification of conservation targets.

## Introduction

Interspecific hybridization is considered to be among the major forces of plant evolution (Arnold 1997). If successful, hybridization facilitates the establishment of new genotypes by combining previously isolated gene pools, and results in significant shifts of allele frequencies at the population level, and may promote advantageous mutations (Barton and Hewitt 1989; Barton 2001; Soltis and Soltis 2009; Soltis 2013). By introgression (backcrossing) of genes and genomes across species barriers, hybridization may lead to establishment of new species and eventually to the collapse of the parental taxa. It may also affect local adaptation and selection processes (Mallet 2008). In general, intermediate characters are usually expected in hybrids, but hybrids often exhibit extreme phenotypes or novel characters, referred to as either ‘heterosis’ or ‘transgressive segregation’ (Rieseberg *et al.* 1999).

Hybridization frequently occurs between taxa of the same ploidy level (Chapman and Abbott 2010), while heteroploid crosses often result in the production of sterile anorthoploid offspring (Köhler *et al.* 2010). However, in both cases, sterility in hybrids can be overcome by polyploidization (producing allopolyploids), which possibly enhances the evolutionary novelty (Finigan *et al.* 2012) and usually results in speciation because hybridization with the parental species produces unviable or sterile offspring (Seehausen 2004, but see also Slotte *et al.* 2008). Consequently, the complex nature of reticulate evolutionary processes complicates the definition of evolutionary and taxonomic units. Moreover, from a conservation point of view, backcrossing of rare hybrid taxa can have a detrimental effect when they are surrounded by larger populations of parental taxa and/or related congeners with incomplete breeding barriers (Allendorf *et al.* 2001).

Reticulate evolution (i.e. introgression and hybridization events, and polyploidization) is considered to be an important mechanism in the speciation of polymorphic *Campanula* section *Heterophylla*, commonly called the ‘harebells’ (Mansion *et al.* 2012; Nicoletti *et al.* 2014). It is a northern hemispheric group consisting of 48 accepted species, nine subspecies and six natural hybrids (The Plant List 2013). The group is taxonomically challenging due to the high overlap of morphological characters and lacks environmentally stable qualitative characters that can be used to differentiate between particular taxa (Shetler 1982). Instead, quantitative vegetative and floral characters are used, e.g. length and shape of corolla and calyx teeth, height of stem and inflorescence, number of flowers per inflorescence, length and width of leaves or form of the root system (Podlech 1965; Shetler 1982). Interestingly, a molecular phylogeny based on the chloroplast *pet*D intron does not support the monophyly of the group (Borsch *et al.* 2009; Mansion *et al.* 2012). Within *Campanula* sect. *Heterophylla* only *C. rotundifolia* and its closest relatives such as *C. baumgartenii* and *C. scheuchzeri* form a monophyletic group. Taxa such as the dwarf alpine species *C. cochleariifolia* and *C. caespitosa* or *C. excisa* form sister clades, but there are also clades in which the members of *Campanula* sect. *Heterophylla* are mixed with other taxa (Mansion *et al.* 2012). Recent introgression and hybridization events together with incomplete lineage sorting are the likely causes for this pattern (Nicoletti *et al.* 2014). Multiple hybridization and allopolyploidization events are assumed (Podlech 1965), with three dominant ploidy levels observed within the group: 2*n = *2*x = *34, 2*n = *4*x = *68 and 2*n = *6*x = *102 (Gadella 1962). Odd ploidy levels (2*n = *3*x = *51, 2*n = *5*x = *85) and aneuploid individuals were only rarely found in nature, but were observed in cultivation after artificial crosses (Podlech 1965; Kovanda 1966; Shetler 1982).

**Figure 1.**
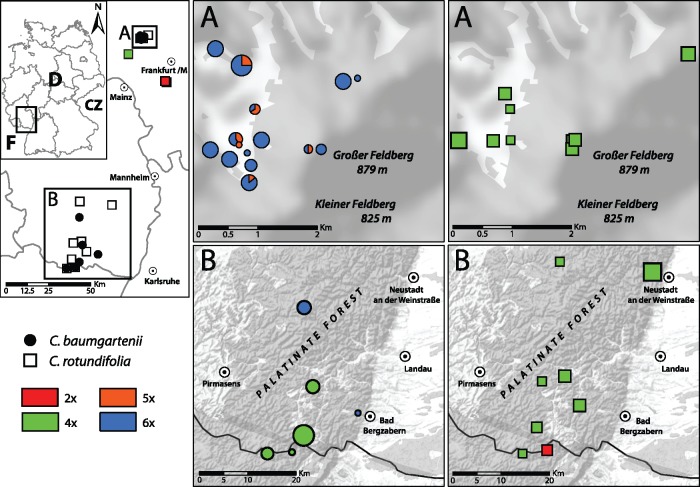
Maps showing the sampling localities of *C. baumgartenii* (circles) and *C. rotundifolia* (squares) in the Taunus (A) and Palatinate Forest (B). Color codes differentiate the DNA-ploidy levels and size of symbols is proportional to the number of analyzed samples. Top left is the study area localized within Germany (D, Germany; F, France; CZ, Czech Republic) as well as in more detail within the German states of Hesse (A) and Rheinland-Palatinate (B).

*Campanula baumgartenii* is a member of *Campanula* sect. *Heterophylla* with a very limited bicentric (disjunct) distribution (Buttler 2002*a*). The very small northern distribution centre comprises the plateau and the west-facing slopes of the Großer Feldberg (Taunus) near Frankfurt am Main, Hesse (Hessen), Germany. The southern distribution centre reaches from the southern part of the Palatinate Forest (Pfälzer Wald), Germany, to the Vosges Mountains, France (Fig. 1) (Buttler and Hodvina 2002; Buttler 2002*b*). In the Palatinate Forest, the distribution of *C. baumgartenii* is continuous with scattered occurrences in the Vosges Mountains. Mainly due to its restricted distribution in Hesse, it is considered nearly threatened (Hemm 2008). A detailed history of the discovery and naming of *C. baumgartenii* can be found in Buttler (2002*a*). In contrast to morphologically closely related and sympatric *C. rotundifolia*, which has prostrate to erect and round stems, *C. baumgartenii* has strictly upright and angular stems. The stem leaves of *C. baumgartenii* are lanceolate and straight while they are narrow lanceolate and often curly in *C. rotundifolia* (Becker 1827). *C. baumgartenii* forms stolons (<0.4 m) and therefore is presumed to be clonal in contrast to the caespitose growth form of *C. rotundifolia* (Buttler 2002*a*). Other qualitative differences are the position of the flower buds before opening (flower buds nodding in *C. baumgartenii*, vs. upright in *C. rotundifolia*) and the absence of short pubescent hairs on *C. baumgartenii* stems (Buttler 2002*a*). Contrary to the rest of the *C*. sect. *Heterophylla*, *C*. *baumgartenii* is self-pollinating (Podlech 1965). Concerning the ploidy levels, tetraploids were previously reported for *C. baumgartenii* originating from the southern part of the southern distribution (Löve and Löve 1961; Podlech 1962) and diploid and tetraploid chromosome counts were reported for *C. rotundifolia* from the adjacent areas of Baden-Württemberg (Kovanda 1966, 1970).

Considering the overall morphological resemblance to *C. rotundifolia* and the peculiar distribution pattern of *C. baumgartenii*, this study aimed to (i) investigate the relationship between these taxa, (ii) elucidate the evolutionary mechanisms and (iii) clarify the taxonomic status of *C. baumgartenii*. We used morphometric analyses, chromosome counting, flow cytometric ploidy estimation, amplified fragment length polymorphism (AFLP) and sequencing of a nuclear and a chloroplast marker. Some closely related taxa of the section *Heterophylla* were also included to assess their potential evolutionary contribution.

## Methods

### Plant material

Plant material was collected from 34 localities in Germany, two localities in France and three localities in Austria. At each locality, 1–12 samples were taken depending on the size of the population, covering the whole distribution range of *C. baumgartenii. C. baumgartenii* and sympatrically co-occurring *C. rotundifolia* were collected at 23 localities in the Taunus and at 11 localities in the Palatinate Forest and Northern Vosges. Populations with a sympatric co-occurrence of both taxa within a distance of 25 m were sampled at five localities in the Taunus (TAU7, TAU12, TAU15, TAU19, TAU21; Table 1) and at one locality in the Northern Vosges (PAL8; Table 1). *C. rotundifolia* was also collected from three additional localities outside the bicentric distribution range of *C. baumgartenii* (Fig. 1). Two individuals (CSK2, population X_TAU12; CAMP01, population CB_PAL12) were transplanted and cultivated in the Botanical Garden, Frankfurt am Main for chromosome counting. Additionally, two common alpine species of *Campanula* sect. *Heterophylla*, *C. scheuchzeri* and *C. cochleariifolia*, were collected from three localities in the Austrian Alps, representing the outgroup from either the same clade or the sister clade respectively (Mansion *et al.* 2012). Leaf samples were immediately dried with silica gel. At most of the collection sites, one herbarium specimen was taken and deposited in Herbarium Senckenbergianum (FR). Detailed collection history is given in Table 1 and **Supporting Information—Table S1**. We used ArcGIS v9.1 (ESRI, USA) software with the Hillshade WMS-layer (Auer *et al.* 2009) and shapefile ‘natural’ from OpenStreetMap (www.openstreetmap.org (15 December 2015)) to present the geographical data.

**Table 1.** Sampling localities.

Taxon	PopID	Sampling Locality and State/Department	Coordinates [WGS 84]	Altitude [m]	Ploidy	Nb
***C. baumgartenii***							
	CB_TAU2	Oberreifenberg, HE	N50.24256 E8.46822	700	6*x*	1/1/1/1/1
	CB_TAU3	Oberreifenberg, HE	N50.24224 E8.46501	690	6*x*	1/7/7/7/7
	CB_TAU5	Oberreifenberg, HE	N50.23214 E8.44288	690	6*x*	1/1/1/1/1
	CB_TAU6	Oberreifenberg, HE	N50.22960 E8.44176	700	6*x*	1/4/4/4/4
	CB_TAU7	Oberreifenberg, HE	N50.22802 E8.44115	700	6*x*	1/6/6/6/6
	CB_TAU9	Niederreifenberg, HE	N50.23182 E8.43535	640	6*x*	1/7/7/7/7
	CB_TAU11	Niederreifenberg, HE	N50.23339 E8.43257	620	6*x*	1/7/7/7/7
	CB_TAU12	Oberreifenberg, HE	N50.24577 E8.44140	630	6*x*	1/9/9/9/8
	CB_TAU13	Oberreifenberg, HE	N50.24857 E8.43552	580	6*x*	1/7/7/7/6
	CB_TAU15	Oberreifenberg, HE	N50.23902 E8.44377	660	6*x*	0/1/1/1/1
	CB_TAU16	Oberreifenberg, HE	N50.23430 E8.44480	680	6*x*	1/7/7/7/7
	CB_TAU19	Oberreifenberg, HE	N50.23471 E8.43876	640	6*x*	1/3/3/3/3
	CB_TAU21	Großer Feldberg, HE	N50.23231 E8.45770	870	6*x*	0/1/1/1/1
	CB_TAU22	Großer Feldberg, HE	N50.23223 E8.45865	870	6*x*	1/3/3/3/2
	CB_PAL1	Hauenstein, RP	N49.16244 E7.84696	400	4*x*	1/5/5/5/5
	CB_PAL3	Johanniskreuz, RP	N49.30982 E7.83650	550	6*x*	1/5/5/5/5
	CB_PAL6	Gimbelhof, BR	N49.04753 E7.77632	330	4*x*	1/1/1/1/1
	CB_PAL8	Wengelsbach, BR	N49.04404 E7.70670	220	4*x*	1/4/4/4/4
	CB_PAL9	Nothweiler, RP	N49.07323 E7.81181	260	4*x*	1/12/12/12/12
	CB_PAL12	Blankenborn, RP	N49.11210 E7.96737	212	6*x*	0/1C/0/0/0
***C. baumgartenii* × *rotundifolia***							
	X_TAU4	Oberreifenberg, HE	N50.23382 E8.43926	650	5*x*	1/1/1/1/1
	X_TAU7	Oberreifenberg, HE	N50.22802 E8.44115	700	5*x*	0/1/1/1/1
	X_TAU12	Oberreifenberg, HE	N50.24577 E8.44140	630	5*x*	0/3C/3/3/3
	X_TAU15	Oberreifenberg, HE	N50.23902 E8.44377	660	5*x*	1/2/2/2/2
	X_TAU19	Oberreifenberg, HE	N50.23471 E8.43876	640	5*x*	0/2/2/2/2
	X_TAU21	Großer Feldberg, HE	N50.23231 E8.45770	870	5*x*	0/1/1/1/1
***C. rotundifolia***							
	CR_TAU1	Oberreifenberg, HE	N50.24537 E8.48662	680	4*x*	1/4/4/4/4
	CR_TAU8	Großer Feldberg, HE	N50.23377 E8.45756	870	4*x*	1/1/1/1/1
	CR_TAU10	Niederreifenberg, HE	N50.23489 E8.43120	620	4*x*	1/5/5/5/5
	CR_TAU14	Oberreifenberg, HE	N50.24138 E8.44286	680	4*x*	1/3/3/3/3
	CR_TAU15	Oberreifenberg, HE	N50.23902 E8.44377	660	4*x*	0/1/1/1/1
	CR_TAU17	Oberreifenberg, HE	N50.23433 E8.44333	670	4*x*	1/1/1/1/1
	CR_TAU18	Oberreifenberg, HE	N50.23441 E8.43925	640	4*x*	1/3/3/3/3
	CR_TAU20	Niedernhausen, HE	N50.15655 E8.32563	280	4*x*	1/5/5/5/5
	CR_TAU21	Großer Feldberg, HE	N50.23231 E8.45770	870	4*x*	1/3/3/3/3
	CR_TAU23	Großer Feldberg, HE	N50.23366 E8.45856	870	4*x*	1/3/3/3/3
	CR_PAL2	Hauenstein, RP	N49.18225 E7.83967	230	4*x*	1/3/3/3/3
	CR_PAL4	Hochspeyer, RP	N49.39450 E7.84658	400	4*x*	1/1/2/2/2
	CR_PAL5	Vorderweidenthal, RP	N49.12617 E7.87594	220	4*x*	1/3/3/3/3
	CR_PAL6	Gimbelhof, BR	N49.04753 E7.77632	330	2*x*	1/2/2/2/2
	CR_PAL7	Hirschthal, RP	N49.09123 E7.75155	250	4*x*	1/2/2/2/2
	CR_PAL8	Wengelsbach, BR	N49.04404 E7.70670	220	4*x*	0/1/1/1/1
	CR_PAL10	Hinterweidenthal, RP	N49.17550 E7.77465	210	4*x*	1/1/1/1/1
	CR_PAL11	Neustadt/Weinstraße, RP	N49.36398 E8.10652	160	4*x*	1/6/6/6/6
	CR_URR1	Langen, HE	N49.99260 E8.62366	100	2*x*	1/3/3/3/3
	CR_URR2	Langen, HE	N50.00014 E8.61066	100	2*x*	1/3/3/3/3
Additional species							
***C. cochleariifolia***							
	CC_ALP1	Hahntennjoch, TI	N47.29144 E10.66457	1990	2*x*	1/0/2/2/2
	CC_ALP3	Obergurgl, TI	N46.84155 E11.04678	2320	2*x*	1/5/5/5/5
***C. scheuchzeri***							
	CS_ALP1	Hahntennjoch, TI	N47.29144 E10.66457	1990	4*x*	1/3/3/3/3
	CS_ALP2	Obergurgl, TI	N46.84016 E11.03110	2290	4*x*	1/8/8/8/8
	CS_ALP3	Obergurgl, TI	N46.84155 E11.04678	2320	4*x*	1/2/3/3/3

Germany, HE Hesse; RP Rhineland-Palatinate, France, BR Bas-Rhin; Austria, TI Tirol; Nb, number of samples examined with morphometry/flow cytometry (C- chromosomes counted)/AFLPs/chloroplast marker sequence/nuclear marker sequence.

### Morphometric analyses

Morphometric analysis was performed on all 42 herbarium specimens, which included all species studied (Table 1, [**see Supporting Information—Table S1**]). Quantitative (6), qualitative (2) and derived (12) characters were assessed (Table 2). General floral and vegetative characters traditionally used for *Campanula* discrimination as well as characters pointed out by Buttler (2002*a*) were measured and scored. All quantitative characters were measured with a ruler to the nearest 0.5 mm without magnification. Quantitative characters were tested for normality (Shapiro–Wilk test). Subsequently, non-parametric Spearman’s correlation coefficient (rho) was computed for all characters due to departure from a normal distribution [**see Supporting Information—Table S2**]. In order to analyze and display the similarities in the data, a principal components analysis (PCA) based on correlation matrix was performed using the software PAST v3.04 (Hammer *et al.* 2001). Canonical discriminant analysis and classificatory discriminant analysis based on non-parametric k-nearest neighbour approach as implemented in MorphoTools (Koutecký 2015) were carried out to specifically test morphological differentiation between sympatric *C. baumgartenii* and *C. rotundifolia.* For discriminant analyses ‘synpetal corolla length/free calyx lobe length’ (character 11) was removed due to high correlation (rho > 0.95) with ‘total corolla length/total calyx length’ (character 8) (Table 2, [**see Supporting Information—Table S2**]).

**Table 2.** List of the morphological characters and ratios analyzed by means of morphometry and their contributions to the first canonical axis in canonical discriminant analyses (CCA).

No	Character	Unit	CCA
1	Hairs on stem	0: absent, 1: length < 0.5 mm, 2: length > 0.5 mm	**−0.363**
2	Stolons	0: absent, 1: present	**−0.268**
3	Number of leaves at middle of stem within 10 cm stem length	number	0.211
4	Largest leaf width	mm	**−0.335**
5	Number of flowers and buds in inflorescence	number	0.001
6	Free corolla limb length/free corolla limb width	ratio	0.134
7	Synpetal corolla length/free corolla limb length	ratio	−0.020
8	Total corolla length/total calyx length	ratio	0.077
9	Synpetal corolla length/synpetal corolla width	ratio	0.040
10	Total corolla length/free corolla width	ratio	0.101
11	Synpetal corolla length/free calyx lobe length	ratio	
12	Free calyx lobe length/free calyx lobe width	ratio	−0.111
13	Synsepal calyx length/Free calyx lobe length	ratio	0.023
14	Leaf length at middle of stem/leaf width at middle of stem	ratio	**0.499**
15	Stem length/leaf length at middle of stem	ratio	0.161
16	Number of branches	number	0.086
17	Length of uppermost stem leaf	mm	−0.127
18	Width of uppermost stem leaf	mm	**−0.281**
19	Number of stem leaves/(stem length—length of inflorescence)	ratio	0.189
20	Length of inflorescence/(stem length—length of inflorescence)	ratio	0.106

Character 11 was excluded from discriminant analyses due to strong correlation (rho > 0.95) with character 8. Bold font highlights the five most extreme values of CCA.

### Chromosome counting

Actively growing root tips were sampled from individuals transplanted to the Botanical Garden, Frankfurt am Main. Root tips were pre-treated with 2 mM 8-hydroxyquinoline for 4 h at 8 °C and fixed in ice-cold 3:1 ethanol: acetic acid for 24 h. Until further analysis, the root tips were stored in 70 % ethanol. Maceration lasted for 5 min in 1N HCl at 60 °C. Microscopic slides were prepared using the squash and smear method with cellophane replacing the glass covers (Murín 1960) followed by staining with aceto-orcein. Chromosomes were counted and photographed using a light microscope Carl Zeiss Axioskop 2 plus (Carl Zeiss Microscopy, Jena, Germany) equipped with a camera Carl Zeiss AxioCam MRc with a 10 × 100 magnification.

### Flow cytometry

Flow cytometric analyses were carried out for all *C. baumgartenii* and *C. rotundifolia* samples and a subset of the remaining samples representing all collected species to check for DNA-ploidy level variation using a standard Otto protocol (Otto 1990; Doležel *et al.* 2007) and a Cyflow Space cytometer (Partec, Germany). Silica-dried or fresh leaf samples and the standard *Glycine max* cv. ‘Polanka’ (Doležel *et al.* 1994) or *Lycopersicum esculentum* cv. ‘Stupické polní tyčkové rané’ (Doležel *et al.* 1992) were chopped together with a razor blade in 1 mL Otto I buffer (100 mM citric acid, 0.5 % (v/v) Tween 20, pH 2–3) and filtered through Partec CellTrics 30 µm (Partec, Germany). The suspension with isolated nuclei was mixed with 1 mL Otto II buffer (400 mM Na_2_PO_4_ · 12H_2_O, pH 8-9) containing 0.2 % (v/v) mercaptoethanol and 0.04 mg DAPI (4′,6-diamidino-2-phenylindole) and fluorescence intensity of 3000 particles was recorded. Sample/standard fluorescence ratios were calculated from the means of the sample and standard fluorescence histograms. Only histograms with coefficients of variation < 5 % for the G_0_/G_1_ peak of the sample were considered. *L. esculentum* was used as internal standard when the G_0_/G_1_ peak of the 2*x C. rotundifolia* overlapped with *G. max* due to similar genome size values. The sample/standard ratios estimated using *L. esculentum* were adjusted to those using *G. max* by multiplying the values by coefficient of 0.802 which was based on 12 repeats of ratios among the two standards. The DNA-ploidy level was attributed based on the sample/standard ratios of the individuals used for chromosome counting and on previously published chromosome counts for the studied species.

### DNA extraction, AFLP analysis, cpDNA and ITS sequencing

The DNA extraction followed the CTAB protocol (Doyle and Doyle 1987) with some modifications. Dried leaf material (10–12 mg) was ground into a fine powder and 650 μL extraction buffer (2 % CTAB, 1.4 M NaCl, 0.1 M Tris-HCl (pH 8), 20 mM EDTA, 0.2 % mercaptoethanol) was added. The samples were incubated for 1 h at 60 °C on a shaker. Subsequently 650 μL chloroform/isoamylalcohol (24:1) was added and the samples were shaken vigorously for 10 min. After 15 min centrifugation at 9000 rpm at room temperature, the supernatant was transferred to new tubes. DNA was precipitated by adding 0.6 times the supernatant’s volume of isopropanol. DNA was centrifuged (15 min, 13 000 rpm, 10 °C), washed once with 70 % ethanol and re-eluted in 200 μL TE-buffer. RNA was removed by adding 5 μL RNase (concentration 10 mg/mL) and incubating for 2 h at 37 °C. Remaining polysaccharides were precipitated by adding 10 μL 5 M NaCl and 75 μL 100 % ethanol, mixing well, incubating 10 min on ice and centrifuging 15 min at 9000 rpm and 10 °C. The supernatant was transferred to new tubes, DNA precipitated with 220 μL isopropylalcohol, washed with 70 % ethanol and re-eluted in 50 μL TE-buffer.

The AFLP-analysis followed Vos *et al.* (1995) with modifications as applied by Nierbauer *et al.* (2014). Twenty-seven primer pairs were screened for variability and even distribution over the length range of 110–600 bp. Three primer pairs: *Hind*III-ACA, *Mse*I-CAT; *Hind*III-AAC, *Mse*I-CAT and *Hind*III-AGC, *Mse*I-CGA were selected for further experiments with 177 individuals plus 19 duplicate samples to identify inconsistent markers [**see Supporting Information—Table S1**]. Differentially fluorescence-labelled PCR products (including 10 % sample replicates) and the GS600 LIZ size standard (Applied Biosystems, USA) were multiplexed and the fragments were separated on a 3730 DNA Analyzer (Applied Biosystems). Raw data were visualized and scored using GeneMarker v2.4.2 (Soft-Genetics, USA) and exported as a presence-absence matrix. In order to analyze and display the similarity among the AFLP genotypes, a principal coordinate analysis (PCoA) based on Jaccard distances was performed using software PAST v3.04 (Hammer *et al.* 2001).

The chloroplast intergenic spacer *rpl*32-*trn*L was amplified using primers *trn*L^(UAG)^ (5′-CTG CTT CCT AAG AGC AGC GT-3′) and *rpl*32-F (5′-CAG TTC CAA AAA AAC GTA CTT C-3′) (Shaw *et al.* 2007). In addition the ITS1 and ITS2 regions including the 5.8S rDNA (nuclear ribosomal DNA) as well as parts of the 18S rDNA and 28S rDNA were sequenced using primers F2 (5′-GTG ACG TCG CGA GAA CTC CAC TG-3′) and R1 (5′-GGT AGT CCC GCC TGA CCT GGG-3′) (Muellner *et al.* 2005). The following PCR mix was used for fragment amplification: 1 μL 10× PCR buffer (PeqLab Red, VWR International, USA), 0.8 μL dNTPs [2 mM], 0.8 μL MgCl_2_ [25 mM], 0.4 μL BSA [1 μg/mL], 0.2 μL primer-f [10 pmol/μL], 0.2 μL primer-r [10 pmol/μL], 0.1 μL Taq (PeqLab), 5.3 μL H_2_O, 1.2 μL template DNA [30 ng/μL]. The PCR mix was incubated at 96 °C for 1 min 45 s followed by 25 cycles of 96 °C for 45 s, 48 °C for 45 s, 72 °C for 1 min followed by a final elongation step of 72 °C for 6 min. The PCR product was electrophoretically checked on 1 % agarose gel and each sample was cleaned using a mix of 0.1 μL Exo I (New England Biolabs, USA), 0.5 μL SAP (USB, Affymetrix, USA) and 9.4 μL H_2_O incubating at 37 °C for 20 min and at 80 °C for 20 min before handing the samples to the sequencing laboratory. The cycle sequencing was accomplished on both strands.

All sequences were edited and a consensus of forward and reverse sequences was made using Lasergene SeqMan Pro v7.1 (DNASTAR, USA). The sequences were aligned using Lasergene MegAlign v7.1 (DNASTAR) and the alignments were manually refined using GeneDoc v2.6.002 (Nicholas *et al.* 1997). Phylogenetic relationships among the cpDNA and the nuclear haplotypes were reconstructed using PopART v1.7 (Leigh and Bryant 2015) employing the TCS algorithm (Clement *et al.* 2000).

## Results

### DNA-ploidy estimation

**Figure 2.**
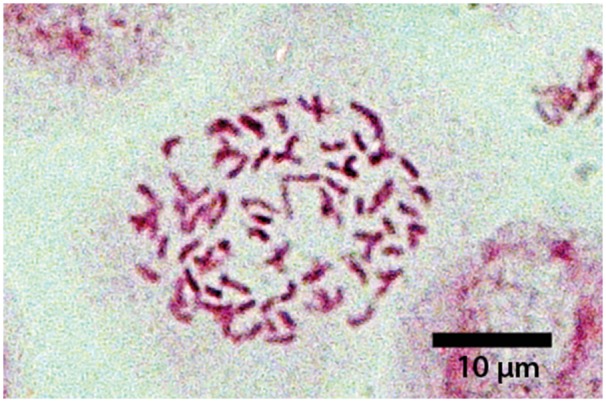
Microphotograph of the somatic chromosomes (2*n* = 5*x* = 85) of *C. baumgartenii* × *rotundifolia*, sample CSK2 from the population X_TAU12.

Altogether, 178 individuals representing four species were studied (Table 3, [**see Supporting Information—Table S1**]). Only one sample/standard ratio, characterized by mean (±SD), was found for each of *C. scheuchzeri* (1.56 ± 0.036, 13 samples) and *C. cochleariifolia* (0.89 ± 0.007, 5 samples), which correspond to previously reported tetraploid and diploid ploidy levels, respectively (Gadella 1964). Two sample/standard ratios were found in *C. baumgartenii* (1.74 ± 0.067, 22 samples; 2.49 ± 0.035, 69 samples) indicating tetraploid (4*x*) and hexaploid (6*x*) DNA-ploidy levels. Hexaploidy was confirmed by chromosome count of the individual CAMP01 (2*n* = 6*x* = 102). Tetraploid *C. baumgartenii* was found in the Palatinate Forest only, while hexaploid individuals occurred in both distribution centres. Two sample/standard ratios were found for *C. rotundifolia* (0.90 ± 0.024, 8 samples; 1.74 ± 0.023, 46 samples) indicating diploid (2*x*) and tetraploid (4*x*) DNA-ploidy levels and corresponding to the previously reported counts from the adjacent area of Baden-Württemberg (Kovanda 1966, 1970). Diploid *C. rotundifolia* was found in the Upper Rhine Rift (populations CR_URR1, CR_URR2) and at one locality in the Palatinate Forest (population CR_PAL6) while tetraploid individuals occurred in the Palatinate Forest and in sympatry with *C. baumgartenii* in the Taunus (Fig. 1A and B). Individuals with a sample/standard ratio indicating a pentaploid (5*x*) DNA-ploidy level (2.12 ± 0.036, 10 samples) occurred at six localities only in the Taunus and were assigned to the natural hybrid *C. baumgartenii* × *rotundifolia* (Table 3). In five localities (TAU7, TAU12, TAU15, TAU19, TAU21) hybrids were found in direct sympatry with *C. baumgartenii*, while only in two localities (TAU15, TAU21) were they found in direct sympatry with *C. rotundifolia*. However, both species are relatively common in this area, so the distance to either of the crossing partners is unlikely to exceed 100 m. Pentaploidy was confirmed by a chromosome count of individual CSK2 (2*n* = 5*x* = 85; Fig. 2).

**Table 3.** DNA-ploidy of studied taxa, material: f, fresh; s, preserved in silica gel.

Species	Samples	Material	Sample/standard ratio ± SD	DNA-ploidy
*C. baumgartenii*	22	s	1.74 ± 0.067	4*x*
*C. baumgartenii*	1/69	f/s	2.49 ± 0.035	6*x*
*C. baumgartenii* × *rotundifolia*	10	s	2.12 ± 0.036	5*x*
*C. rotundifolia*	8	s	0.90 ± 0.024	2*x*
*C. rotundifolia*	46	s	1.74 ± 0.032	4*x*
*C. scheuchzeri*	10/3	f/s	1.56 ± 0.036	4*x*
*C. cochleariifolia*	5	f	0.89 ± 0.007	2*x*

### Morphometric analyses

**Figure 3.**
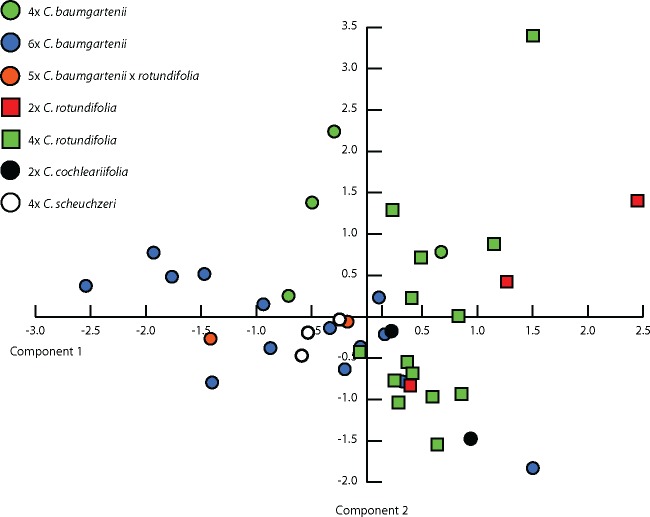
PCA of 20 vegetative, floral and derived characters of 42 herbarium specimens of *C. baumgartenii*, *C. rotundifolia*, *C. scheuchzeri* and *C. cochleariifolia*. The first two axes explained 24.8 and 16.3 % of the total variation.

**Figure 4.**
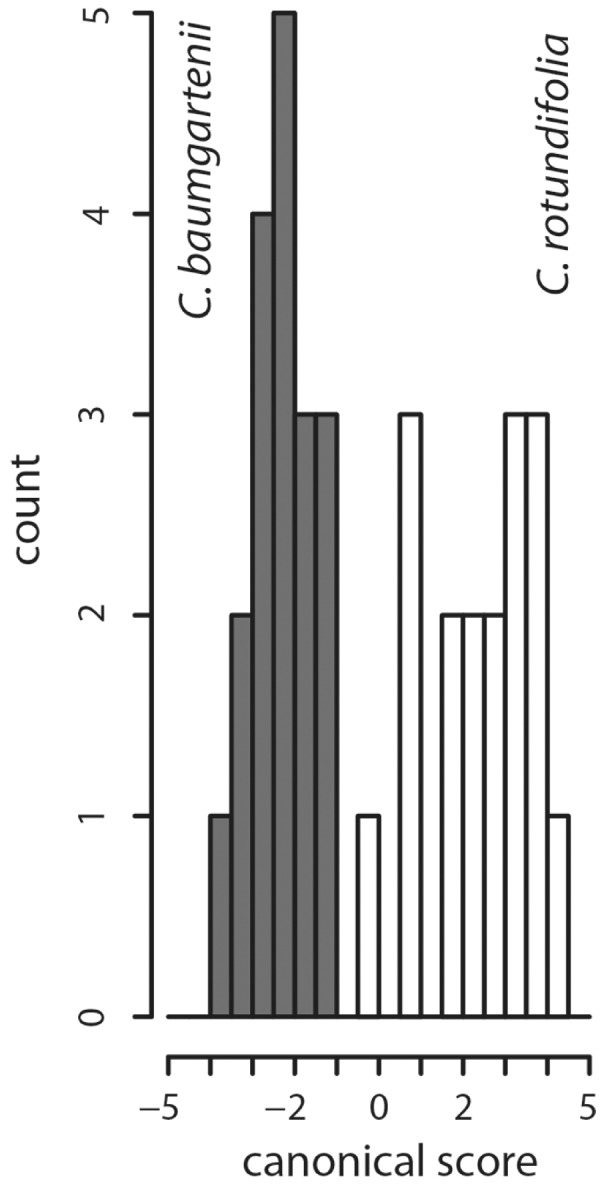
Histogram of canonical scores of 19 vegetative, floral and derived characters for studied *C. baumgartenii* and *C. rotundifolia* individuals.

The PCA (Fig. 3) based on morphological data separated 6*x C. baumgartenii*, 4*x C. baumgartenii*, 2*x C. rotundifolia* and *C. cochleariifolia*. Interestingly, *C. scheuchzeri* and 5*x C. baumgartenii* × *rotundifolia* cluster with 6*x C. baumgartenii*. The 4*x C. rotundifolia* group overlaps with 6*x C. baumgartenii*, 4*x C. baumgartenii* and 2*x C. rotundifolia.* The first axis explained 24.8 % of the total variation and was most strongly correlated with presence of stolons (character 2; Table 2), width of uppermost stem leaf and length of uppermost stem leaf (characters 17, 18). The second axis explained 16.3 % of the total variation and was most strongly correlated with the number of flowers (character 5), number of branches (character 16) and length of the inflorescence divided by the difference between stem length and length of the inflorescence (character 20). When specifically tested for morphological differentiation between sympatric *C. baumgartenii* and *C. rotundifolia* the overall predictive accuracy of the canonical discriminant analysis was 100 % (Fig. 4). Characters best discriminating between the groups were ‘leaf length at middle of stem/leaf width at middle of stem’ (character 14), ‘presence of stem hairs’ (character 1) and ‘largest leaf width’ (character 4; Table 2). These results were also confirmed by classificatory discriminant analysis based on non-parametric k-nearest neighbour approach with lower, but still adequate, overall predictive accuracy of 91.4 %.

### AFLP analysis

**Figure 5.**
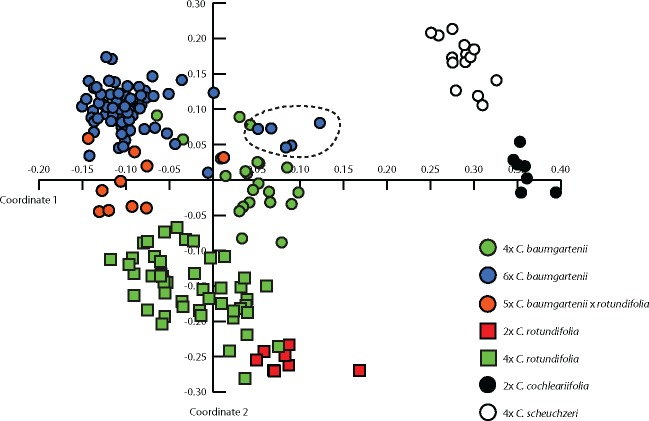
PCoA of AFLP genotypes of 177 samples of *C. baumgartenii*, *C. rotundifolia*, *C. scheuchzeri* and *C. cochleariifolia* using Jaccard distances. The first two axes explained 10.9 and 10.0 % of the total variation. Dotted line marks the 6*x C. baumgartenii* from Palatinate Forest.

In total, 316 fragments were generated for 177 samples. After removing fragments with an error rate of more than 10 %, 304 fragments remained out of which 289 (95.1 %) were polymorphic. The average error rate over all samples was 1.8 %. Private markers were found in Taunus 6*x C. baumgartenii* (4), *C. scheuchzeri* (1) and *C. cochleariifolia* (5). The remaining markers (96.5 %) were shared among the species. Two-dimensional PCoA based on Jaccard distances clearly separated *C. baumgartenii and C. rotundifolia* as well as genetically more distant *C. scheuchzeri* and *C. cochleariifolia* (Fig. 5)*.* The ploidy levels within the taxa (4*x*, 6*x C. baumgartenii*, 2*x*, 4*x C. rotundifolia*) also formed partially overlapping separated clusters. The 5*x C. baumgartenii* × *rotundifolia* individuals were found in an intermediate position between the parental taxa. However, the first two axes explained only 21 % of the overall variation.

### cpDNA sequence data

Sequence length of the *rpl*32-*trn*L intron ranged from 779 to 894 bp. Interestingly, a species-specific length of this fragment was recorded: *C. baumgartenii* (4*x*, 6*x*) with 836 bp, 4*x C. rotundifolia* with 809 bp, 2*x C. rotundifolia* with 779 bp, 4*x C. scheuchzeri* with 831 bp, 2*x C. cochleariifolia* with 890 bp. Only very few deviations from the typical lengths were recorded concerning mainly *C. rotundifolia*, but also one *C. baumgartenii* (CNO10, [**see Supporting Information—Table S1**]) with a 55 bp gap at positions 709–763 which is otherwise only present in *C. rotundifolia* and *C. cochleariifolia*. In total 8 out of the 10 pentaploid *C. baumgartenii* × *rotundifolia* hybrids had the *rpl*32-*trn*L intron sequence length of *C. baumgartenii* and two had the sequence length of tetraploid *C. rotundifolia*. The total length of the alignment was 982 bp. A stretch of 68 bp between positions 426–493 was excluded from further analysis since it could not be aligned meaningfully. The length of the remaining alignment used for the final analysis was 914 bp.

**Figure 6.**
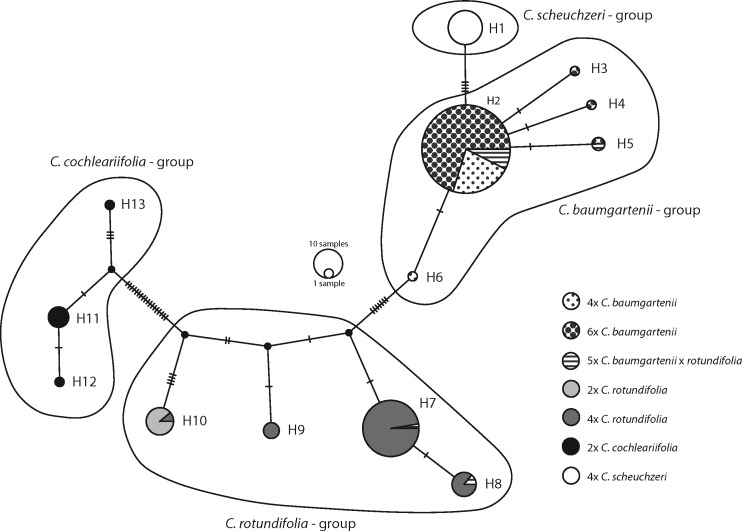
Haplotype network of the cpDNA *trn*L-*rpl*32 intron of *C. baumgartenii*, *C. rotundifolia*, *C. scheuchzeri* and *C. cochleariifolia.* Small bars indicate the number of mutational steps/coded indels necessary to link the haplotypes.

The cpDNA haplotype network recovered 13 haplotypes (H1-H13, Fig. 6), which could be divided into four haplotype groups: *C. scheuchzeri*, *C. baumgartenii*, *C. rotundifolia* and *C. cochleariifolia* group. All studied *C. scheuchzeri* shared one haplotype (H1). It is separated by five mutational steps from the *C. baumgartenii* group comprising haplotype H2 retrieved from the majority of 4*x* and 6*x C. baumgartenii* as well as 5*x C. baumgartenii* × *rotundifolia* hybrids. A few *C. baumgartenii* and *C. baumgartenii* × *rotundifolia* individuals share haplotypes H3-H6 deviating by two mutational steps from H2. The *C. rotundifolia* haplotype group is separated by eight mutational steps from the main *C. baumgartenii* group comprising haplotypes H7-H10. The majority of the 4*x C. rotundifolia* and one 5*x C. baumgartenii* × *rotundifolia* individuals share haplotype H7 with additional samples in up to six mutational steps distance. Only a single 4*x C. rotundifolia* individual shares the haplotype (H10) with the 2*x C. rotundifolia* individuals, which are 12 mutational steps from H7. Finally, the haplotypes of *C. cochleariifolia* (H11–H13) form a group separated by 24 mutational steps from 2*x C. rotundifolia* (H10). Since chloroplasts are maternally inherited in the Campanulaceae (Harris and Ingram 1991), the strongly deviating cpDNA haplotypes of *C. baumgartenii* and *C. rotundifolia* allowed the determination of the direction of the cross in the 10 detected pentaploid hybrids. Hence, *C. baumgartenii* was the maternal parent in eight cases and the pollen parent in two.

### ITS sequence data

**Figure 7.**
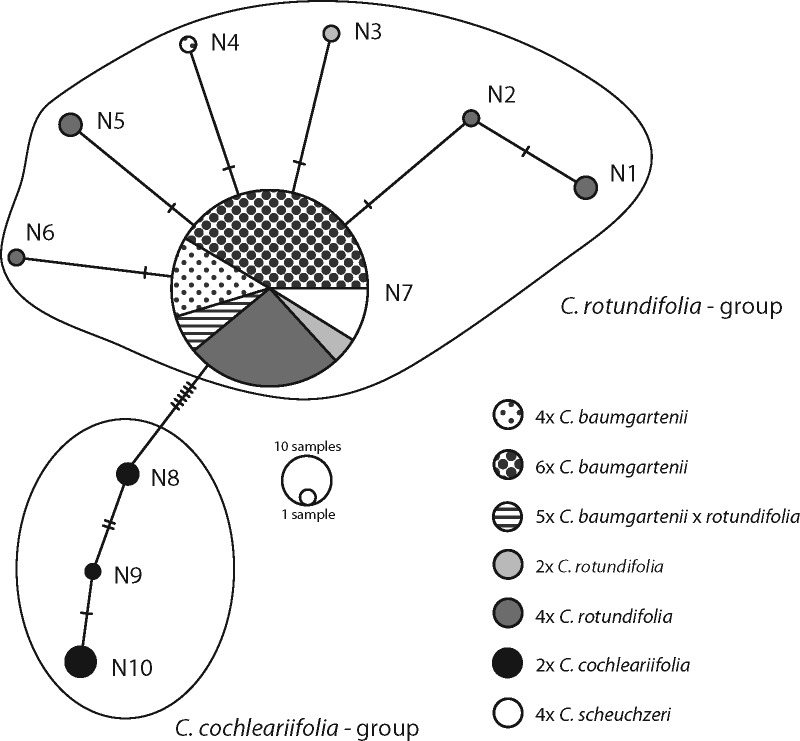
Haplotype network of the ITS region comprising ITS1, 5.8S rDNA, ITS2 and parts of 18S rDNA and 28S rDNA of *C. baumgartenii*, *C. rotundifolia*, *C. scheuchzeri* and *C. cochleariifolia.* Small bars indicate the number of mutational steps/coded indels necessary to link the haplotypes.

The length of the fragment comprising ITS1, 5.8S rDNA and ITS2 as well as parts of 18S rDNA and 28S rDNA was 760 bp for all studied taxa. Most of the individuals (89.3 %) of *C. baumgartenii* (both 4*x* and 6*x*), *C. rotundifolia* (4*x*) as well as *C. baumgartenii* × *rotundifolia* (5*x*) showed several sites with ambiguous bases (Table 4). These sites were however treated as gaps since the different alleles could not be reconstructed. The ITS network recovered 10 ribotypes (N1-N10, Fig. 7) separating two ribotype groups. *C. baumgartenii*, *C. rotundifolia* and *C. scheuchzeri* form one group (N1–N7) which is separated by seven mutational steps from *C. cochleariifolia* (N8–N9). Most of the *C. rotundifolia*, all *C. scheuchzeri* and *C. baumgartenii* individuals (except one) share the same ITS ribotype (N7). *C. rotundifolia* (2*x* and 4*x*) shows some variation in the ITS sequence but has always very few mutational steps distance from N7 (N1–N6).

**Table 4.** Positions in the 760 bp ITS1—5.8S rDNA—ITS2 fragment with ambiguous bases.

Taxon	Pos 185	Pos 254	Pos 331	Pos 570	Pos 631	Pos 680
*C. baumgartenii* 6*x*	A, R	C, T, Y	A, R	T, Y	A, W	A, R
*C. baumgartenii* 4*x*	A, R	T, Y	A	T, Y	A, W	A, R
*C. baumgartenii* × *rotundifolia* 5*x*	A, R	C, T, Y	A, R	T, Y	A, W	R
*C. rotundifolia* 4*x*	A, G, R	C, T, Y	A, G, R	C, T, Y	A, T, W	A, G, R
*C. rotundifolia* 2*x*	G	C	A	C, T	T	G
*C. cochleariifolia* 2*x*	G	C	A	T	A	G
*C. scheuchzeri* 4*x*	G	C	A	C	A	G

## Discussion

### Taxonomic status of *C. baumgartenii*

Exploratory PCA based on morphometric data (Fig. 3) confirmed that morphological differences in *Campanula* sect. *Heterophylla* have not yet accumulated to a reliable and measurable degree due to presumed recent origins of particular taxa (Kovacić and Nikolić 2006; Mansion *et al.* 2012; Nicoletti *et al.* 2014). The characters that contributed most to the variation of the PCA (petal-width, width of uppermost stem leaf, calyx-width, number of branches, number of stem leaves, length of the inflorescence) are either individually not differentiating enough or could be considered ecologically unstable (Buttler 2002*a*) for distinction between *C. baumgartenii* and *C. rotundifolia*. Nevertheless, when morphological differentiation between *C. baumgartenii* and *C. rotundifolia* was specifically tested using discriminant approaches, these taxa were separated accurately (Fig. 4). Characters previously proposed by Buttler (2002*a*) (broad straight leaves, type of hairs on stem and the presence of stolons) revealed the highest contributions to the first canonical axis in canonical discriminant analyses (Table 2). Genetic markers also strongly support the distinction between these two lineages. The AFLP based PCoA differentiated *C. baumgartenii* and *C. rotundifolia* (Fig. 5) as well as highly divergent cpDNA haplotypes (Fig. 6). Additionally, the occurrence of 6*x* cytotype (see also below) only in *C. baumgartenii* further support the divergence between the two species. Hence, the results support the maintenance of *C. baumgartenii* as a separate species.

### Geographical distribution and cytotype differentiation

This study reveals the occurrence of cytogenetic diversity in the rare endemic *C. baumgartenii*. In previous studies only 4*x* individuals of *C. baumgartenii* were recognized (Löve and Löve 1961; Podlech 1962) because the studied material originated only from the southern part of the southern distribution centre. This study complements previous results, reporting the occurrence of an additional ploidy level (6*x*) that seems to be more frequent than the 4*x* previously described, and gives a full picture of the species’ cytogeography revealing a bicentric distribution.

Disjunct distributions in central European non-apomictic plant species are very rare (e.g. Szövényi *et al.* 2009; Jiménez-Mejías *et al.* 2015) and could be explained by (i) different evolutionary histories, (ii) colonization from different glacial refugia, (iii) representing a remainder of a once continuous distribution area or (iv) dispersal events. The chloroplast haplotype sharing (H2) and the presence of 6*x C. baumgartenii* populations in the Taunus and the Palatinate Forest, suggests that the peculiar bicentric distribution of this species would likely be a remainder of a once continuous distribution area or the result of a dispersal event. Unfortunately, the relatively close geographical distance does not allow to distinguish between these two processes.

AFLP based PCoA recovered a certain genetic differentiation among the cytotypes. This pattern is quite common when studying polyploid complexes due to an effect of ploidy itself (i.e. genomes of different sizes), which usually results in a different number of AFLP fragments (e.g. Balao *et al.* 2010; Ma *et al.* 2010; Paule *et al.* 2012). Limited gene flow due to the presence of two different cytotypes, together with the disjunct distribution of *C. baumgartenii* cytotypes might additionally contribute to the differentiation. However, PCoA recovered also an overlap between 4*x* and 6*x C. baumgartenii* from Palatinate Forest. The obtained result enables us to propose two hypotheses about the origin of differential ploidy levels and an ancestral cytotype within *C. baumgartenii*. On the one hand, AFLP clustering of 6*x C. baumgartenii* from Palatinate Forest (population CB_PAL3) with 4*x C.baumgartenii* suggests that 6*x* mothers (shared presence of the haplotype H2) expanded or dispersed from the Taunus to Palatinate Forest where they could have hybridized with 2*x C. rotundifolia*, also present in the Palatinate Forest (population CR_PAL6), giving rise to 4*x* offspring. On the other hand, 4*x C. baumgartenii* mothers could have hybridized with 2*x C. rotundifolia* in the Palatinate Forest and undergone genome duplication (formation of 6*x* entities) followed by range expansion or stochastic dispersal events to the Taunus. Our data preclude us to be conclusive on the answer to this question. However, the ancestral state of 6*x* cytotype and more recent hybrid origin of 4*x C. baumgartenii* are supported by the fact that the standard deviation of the relative DNA content (sample/standard ratio) of 4*x C. baumgartenii* is about twice as high as for 6*x C. baumgartenii* or for 4*x C. rotundifolia* (Table 3). Such variance reflects active genome reorganization and has been associated with recent hybridization and introgression processes (Hanušová *et al*. 2014). Additionally, the 4*x C. baumgartenii* appears in an intermediate position between 6*x C. baumgartenii and C. rotundifolia* in the AFLP-based PCoA clustering. All these suggest that the scenario of hybrid/introgression origin of 4*x C. baumgartenii* and the ancestral state of 6*x C. baumgartenii* is more likely, although, recent introgression of 4*x C. rotundifolia* might also produce a similar pattern.

### Origin of *C. baumgartenii*

The fertility among species of *C.* sect. *Heterophylla* is high (Shetler 1982) and most of the known natural hybrids in the genus *Campanula* belong to this group (The Plant List 2013). Only different ploidy levels have been suggested to cause some degree of genetic separation (Podlech 1965). Thus, it is likely that gene flow occurs between members of *C.* sect. *Heterophylla* growing in sympatric populations. This would explain the lack of good morphological characters to discern species within *C.* sect. *Heterophylla*. A cross between diploid *C. rotundifolia* and diploid *C. serrata* with subsequent polyploidization was suggested to have led to the origin of *C. baumgartenii* (Podlech 1965). However, according to Mansion *et al.* (2012)*C. serrata* has a divergent *petD* sequence, which excludes it as the maternal parent of *C. baumgartenii*. Additionally, *C. serrata* is endemic to the Carpathians, which makes it unlikely to be the parent of a species that occurs north-west of the Alps.

Nevertheless, our data point towards a hybrid origin of *C. baumgartenii* mainly due to the presence of ITS base ambiguities (Table 4, Calonje *et al.* 2009), and divergent cpDNA haplotypes. Similar morphology (Fig. 3), close–molecular relationship (Fig. 5), and shared ITS ribotype (Fig. 7) suggest that the widely distributed *C. rotundifolia* is one of the parental species. A second parental taxon could be deduced from the divergent cpDNA haplotypes, which might be related to *C. scheuchzeri* (Fig. 6). In fact, *C. scheuchzeri* has a small population in the southern Black Forest (Aichinger 1957) and was likely distributed further north in postglacial times. Interestingly, 6*x C. baumgartenii* was in the Taunus found only in localities above 500 m altitude, a fact that might support the introgression with a cold adapted species. Alternatively, niche differentiation of the 6*x C. baumgartenii* vs. 4*x C. rotundifolia* and self-pollinating reproduction mode reported for 6*x C. baumgartenii* (Podlech 1965) might have also been a way for escaping the minority cytotype exclusion (Levin 1975).

### Natural hybrid *C. baumgartenii* × *rotundifolia*

*Campanula* sect. *Heterophylla* hybrids with an uneven set of chromosomes are considered to be rare in nature (Kovanda 1977). However, in the Taunus 5*x C. baumgartenii* × *rotundifolia* individuals were found with a relatively high frequency in an area that can be considered as one large sympatric population of the two parental species. Hybrid origin of these pentaploids was supported by the intermediate position in the AFLP-based PCoA (Fig. 5) as well as by chloroplast haplotype sharing. *C. baumgartenii* × *rotundifolia* revealed haplotypes specific to both presumed parents, thus both *C. baumgartenii* and *C. rotundifolia* could be considered maternal parents. The existence of *C. baumgartenii* × *rotundifolia* was previously suggested, because plants with intermediate characters were found in sympatric populations of the parental species (Buttler 2002*a*).

Anecdotal observations from a 5*x C. baumgartenii* × *rotundifolia* (CSK2) transplanted to the Frankfurt Botanical Garden suggest that pentaploids are not sterile since it produced numerous capsules containing seeds with well-developed endosperm (K. U. Nierbauer, pers. obs.). The presence of the pentaploids, introgressed by one of the parental species, raises some additional conservation issues for already nearly threatened *C. baumgartenii* (IUCN category: NT). Gene swamping, production of hybrid seeds at the expense of conspecific seeds and/or hybrid competition for abiotic or biotic resources may eventually have detrimental impacts in the parental taxa (Allendorf *et al.* 2001). A detailed study of the fertility and competitiveness of 5*x* hybrids would help to guide conservation measures. Nevertheless, in future, special conservation focus should be given to both 4*x* and 6*x* plants which bear the *C. baumgartenii* haplotype group (Fig. 6) thus covering the cytogenetic diversity of the species.

## Conclusions

Using a combination of nuclear and chloroplast genetic markers as well as flow cytometric ploidy estimations and morphometric analyses, the differentiation between polyploid *C. baumgartenii* and diploid and tetraploid *C. rotundifolia* was found to be sufficient to support the currently accepted taxonomic ranks. A hybrid origin of *C. baumgartenii* was proposed, with *C. rotundifolia* representing one of the parental taxa. The existence of hybrid pentaploid *C. baumgartenii* × *rotundifolia*, for which both parental taxa served as maternal parents, suggests dynamic ongoing evolutionary processes and might represent a threat to the persistence of rare *C. baumgartenii*. To our knowledge, this is the first study that managed to resolve the relationships among the *C. rotundifolia* and one of its sister species in the *Heterophylla* section on a local scale. This study also exemplifies that detailed population genetic studies can provide a solid basis for taxonomic delimitation within *Campanula* section *Heterophylla* as well as for sound identification of conservation targets.

## Accession Numbers

The sequences are deposited in the NCBI GenBank (KY034455-KY034641, KY009257-KY009449) [**see Supporting Information—Table S1**].

## Sources of Funding

Funding was provided by the Senckenberg Research Institute and Natural History Museum Frankfurt and partially by Deutsche Forschungsgemeinschaft (DFG) in scope of the project “Die karyologische Datenbank zur Flora von Deutschland (Gefäßpflanzen)” (Zi 557/13-1).

## Contributions by the Authors

G.Z., J.P. and K.U.N conceived the ideas and designed the research, K.U.N carried out fieldwork and laboratory analyses, K.U.N. and J.P. performed statistical analysis; J.P and K.U.N. wrote the article. All authors provided comments, read and approved the final version of the article.

## Conflict of Interest Statement

None declared.

## Supplementary Material

Supplementary Data
