# Supplementary material for: Heteroploid reticulate evolution and taxonomic status of an endemic species with bicentric geographical distribution
Source: AoB Plants. 2017 Jan 25;9(1):plx002. doi: 10.1093/aobpla/plx002 (PMC5463600; doi:10.1093/aobpla/plx002)
Supplement: Supplementary Data [file plx002_Supp.doc]

Table S1. List of studied *Campanula* accessions and performed experiments. FCM – flow cytometry (s - silikagel material used for FCM, cc – chromosome counted), AFLP – amplified fragment length polymorphism. Country codes follow ISO 3166-1 Alpha-3. Collector abbreviations: KUN – Kai Uwe Niebauer, TG – Thomas Gregor.

| **Taxon** | **Mat Nr** | **PopID** | | **Locality** | **FCM** | **AFLP** | **cp haplotype** | **GenBank No.** | **ITS ribotype** | **GenBank No.** | **Herbarium No.** |
| --- | --- | --- | --- | --- | --- | --- | --- | --- | --- | --- | --- |
| ***Campanula baumgartenii*** | | | | | | | | | | | |
|  | CSI1 | CB_TAU2 | | DEU, Hesse, Oberreifenberg; KUN | 6x_s | X | H2 | KY034602 | N7 | KY009412 | FR-0121732 |
|  | CSB1  CSB2  CSB3  CSB4  CSB5  CSB6  CSB7 | CB_TAU3  CB_TAU3  CB_TAU3  CB_TAU3  CB_TAU3  CB_TAU3  CB_TAU3 | | DEU, Hesse, Oberreifenberg; KUN  DEU, Hesse, Oberreifenberg; KUN  DEU, Hesse, Oberreifenberg; KUN  DEU, Hesse, Oberreifenberg; KUN  DEU, Hesse, Oberreifenberg; KUN  DEU, Hesse, Oberreifenberg; KUN  DEU, Hesse, Oberreifenberg; KUN | 6x_s  6x_s  6x_s  6x_s  6x_s  6x_s  6x_s | X  X  X  X  X  X  X | H2  H2  H2  H2  H2  H2  H2 | KY034582  KY034583  KY034584  KY034585  KY034586  KY034587  KY034588 | N7  N7  N7  N7  N7  N7  N7 | KY009392  KY009393  KY009394  KY009395  KY009396  KY009397  KY009398 | FR-0121731  -  -  -  -  -  - |
|  | CLI1 | CB_TAU5 | | DEU, Hesse, Oberreifenberg; KUN | 6x_s | X | H2 | KY034537 | N7 | KY009342 | FR-0121729 |
|  | CLI2  CLI3  CLI4  CLI5 | CB_TAU6  CB_TAU6  CB_TAU6  CB_TAU6 | | DEU, Hesse, Oberreifenberg; KUN  DEU, Hesse, Oberreifenberg; KUN  DEU, Hesse, Oberreifenberg; KUN  DEU, Hesse, Oberreifenberg; KUN | 6x_s  6x_s  6x_s  6x_s | X  X  X  X | H2  H2  H2  H2 | KY034538  KY034539  KY034540  KY034541 | N7  N7  N7  N7 | KY009343  KY009344  KY009345  KY009346 | FR-0121728  -  -  - |
|  | CHK1  CHK3  CHK4  CHK5  CHK6  CHK7 | CB_TAU7  CB_TAU7  CB_TAU7  CB_TAU7  CB_TAU7  CB_TAU7 | | DEU, Hesse, Oberreifenberg; KUN  DEU, Hesse, Oberreifenberg; KUN  DEU, Hesse, Oberreifenberg; KUN  DEU, Hesse, Oberreifenberg; KUN  DEU, Hesse, Oberreifenberg; KUN  DEU, Hesse, Oberreifenberg; KUN | 6x_s  6x_s  6x_s  6x_s  6x_s  6x_s | X  X  X  X  X  X | H2  H2  H4  H2  H2  H2 | KY034496  KY034498  KY034499  KY034500  KY034501  KY034502 | N7  N7  N7  N7  N7  N7 | KY009297  KY009299  KY009300  KY009301  KY009302  KY009303 | FR-0121727  -  -  -  -  - |
|  | CHT1  CHT2  CHT3  CHT4  CHT5  CHT6  CHT7 | CB_TAU9  CB_TAU9  CB_TAU9  CB_TAU9  CB_TAU9  CB_TAU9  CB_TAU9 | | DEU, Hesse, Niederreifenberg; KUN  DEU, Hesse, Niederreifenberg; KUN  DEU, Hesse, Niederreifenberg; KUN  DEU, Hesse, Niederreifenberg; KUN  DEU, Hesse, Niederreifenberg; KUN  DEU, Hesse, Niederreifenberg; KUN  DEU, Hesse, Niederreifenberg; KUN | 6x_s  6x_s  6x_s  6x_s  6x_s  6x_s  6x_s | X  X  X  X  X  X  X | H2  H2  H2  H2  H2  H2  H2 | KY034505  KY034506  KY034507  KY034508  KY034509  KY034510  KY034511 | N7  N7  N7  N7  N7  N7  N7 | KY009306  KY009307  KY009308  KY009309  KY009310  KY009311  KY009312 | FR-0121736  -  -  -  -  -  - |
|  | CHU1  CHU2  CHU3  CHU4  CHU5  CHU6  CHU7 | CB_TAU11  CB_TAU11  CB_TAU11  CB_TAU11  CB_TAU11  CB_TAU11  CB_TAU11 | | DEU, Hesse, Niederreifenberg; KUN  DEU, Hesse, Niederreifenberg; KUN  DEU, Hesse, Niederreifenberg; KUN  DEU, Hesse, Niederreifenberg; KUN  DEU, Hesse, Niederreifenberg; KUN  DEU, Hesse, Niederreifenberg; KUN  DEU, Hesse, Niederreifenberg; KUN | 6x_s  6x_s  6x_s  6x_s  6x_s  6x_s  6x_s | X  X  X  X  X  X  X | H2  H2  H2  H2  H2  H2  H2 | KY034512  KY034513  KY034514  KY034515  KY034516  KY034517  KY034518 | N7  N7  N7  N7  N7  N7  N7 | KY009313  KY009314  KY009315  KY009316  KY009317  KY009318  KY009319 | FR-0121734  -  -  -  -  -  - |
|  | CSK1  CSK4  CSK5  CSK6  CSK7  CSK9  CSK10  CSK11  CSK12 | CB_TAU12  CB_TAU12  CB_TAU12  CB_TAU12  CB_TAU12  CB_TAU12  CB_TAU12  CB_TAU12  CB_TAU12 | | DEU, Hesse, Oberreifenberg; KUN  DEU, Hesse, Oberreifenberg; KUN  DEU, Hesse, Oberreifenberg; KUN  DEU, Hesse, Oberreifenberg; KUN  DEU, Hesse, Oberreifenberg; KUN  DEU, Hesse, Oberreifenberg; KUN  DEU, Hesse, Oberreifenberg; KUN  DEU, Hesse, Oberreifenberg; KUN  DEU, Hesse, Oberreifenberg; KUN | 6x_s  6x_s  6x_s  6x_s  6x_s  6x_s  6x_s  6x_s  6x_s | X  X  X  X  X  X  X  X  X | H2  H2  H2  H2  H5  H2  H2  H2  H2 | KY034603  KY034606  KY034607  KY034608  KY034609  KY034611  KY034612  KY034613  KY034614 | N7  N7  N7  N7  N7  N7  N7  -  N7 | KY009413  KY009416  KY009417  KY009418  KY009419  KY009421  KY009422  -  KY009423 | FR-0121741  -  -  -  -  -  -  -  - |
|  | CWB1  CWB2  CWB3  CWB4  CWB5  CWB6  CWB7 | CB_TAU13  CB_TAU13  CB_TAU13  CB_TAU13  CB_TAU13  CB_TAU13  CB_TAU13 | | DEU, Hesse, Oberreifenberg; KUN  DEU, Hesse, Oberreifenberg; KUN  DEU, Hesse, Oberreifenberg; KUN  DEU, Hesse, Oberreifenberg; KUN  DEU, Hesse, Oberreifenberg; KUN  DEU, Hesse, Oberreifenberg; KUN  DEU, Hesse, Oberreifenberg; KUN | 6x_s  6x_s  6x_s  6x_s  6x_s  6x_s  6x_s | X  X  X  X  X  X  X | H2  H2  H2  H2  H2  H2  H2 | KY034625  KY034626  KY034627  KY034628  KY034629  KY034630  KY034631 | N7  N7  N7  N7  N7  N7  - | KY009434  KY009435  KY009436  KY009437  KY009438  KY009439  - | FR-0121740  -  -  -  -  -  - |
|  | CSW2 | CB_TAU15 | | DEU, Hesse, Oberreifenberg; KUN | 6x_s | X | H2 | KY034619 | N7 | KY009428 | - |
|  | CSH1  CSH2  CSH3  CSH4  CSH5  CSH6  CSH7 | CB_TAU16  CB_TAU16  CB_TAU16  CB_TAU16  CB_TAU16  CB_TAU16  CB_TAU16 | | DEU, Hesse, Oberreifenberg; KUN  DEU, Hesse, Oberreifenberg; KUN  DEU, Hesse, Oberreifenberg; KUN  DEU, Hesse, Oberreifenberg; KUN  DEU, Hesse, Oberreifenberg; KUN  DEU, Hesse, Oberreifenberg; KUN  DEU, Hesse, Oberreifenberg; KUN | 6x_s  6x_s  6x_s  6x_s  6x_s  6x_s  6x_s | X  X  X  X  X  X  X | H3  H2  H2  H2  H2  H2  H2 | KY034594  KY034595  KY034596  KY034597  KY034598  KY034599  KY034600 | N7  N7  N7  N7  N7  N7  N7 | KY009404  KY009405  KY009406  KY009407  KY009408  KY009409  KY009410 | FR-0121745  -  -  -  -  -  - |
|  | CHW4  CHW6  CHW8 | CB_TAU19  CB_TAU19  CB_TAU19 | | DEU, Hesse, Oberreifenberg; KUN  DEU, Hesse, Oberreifenberg; KUN  DEU, Hesse, Oberreifenberg; KUN | 6x_s  6x_s  6x_s | X  X  X | H2  H2  H2 | KY034522  KY034524  KY034526 | N7  N7  N7 | KY009323  KY009325  KY009327 | FR-0121742  -  - |
|  | CGF2 | CB_TAU21 | | DEU, Hesse, Großer Feldberg; KUN | 6x_s | X | H2 | KY034467 | N7 | KY009269 | - |
|  | CGF6  CGF7  CGF8 | CB_TAU22  CB_TAU22  CB_TAU22 | | DEU, Hesse, Großer Feldberg; KUN  DEU, Hesse, Großer Feldberg; KUN  DEU, Hesse, Großer Feldberg; KUN | 6x_s  6x_s  6x_s | X  X  X | H2  H2  H2 | KY034471  KY034472  KY034473 | N7  -  N7 | KY009273  -  KY009274 | FR-0121771  -  - |
|  | CDE1  CDE2  CDE3  CDE4  CDE5 | CB_PAL1  CB_PAL1  CB_PAL1  CB_PAL1  CB_PAL1 | | DEU, Rhineland-Palatinate, Hauenstein; KUN  DEU, Rhineland-Palatinate, Hauenstein; KUN  DEU, Rhineland-Palatinate, Hauenstein; KUN  DEU, Rhineland-Palatinate, Hauenstein; KUN  DEU, Rhineland-Palatinate, Hauenstein; KUN | 4x_s  4x_s  4x_s  4x_s  4x_s | X  X  X  X  X | H2  H2  H2  H2  H2 | KY034461  KY034462  KY034463  KY034464  KY034465 | N7  N7  N7  N7  N7 | KY009263  KY009264  KY009265  KY009266  KY009267 | FR-0121749  -  -  -  - |
|  | CAB1  CAB2  CAB3  CAB4  CAB5 | CB_PAL3  CB_PAL3  CB_PAL3  CB_PAL3  CB_PAL3 | | DEU, Rhineland-Palatinate, Johanniskreuz; KUN  DEU, Rhineland-Palatinate, Johanniskreuz; KUN  DEU, Rhineland-Palatinate, Johanniskreuz; KUN  DEU, Rhineland-Palatinate, Johanniskreuz; KUN  DEU, Rhineland-Palatinate, Johanniskreuz; KUN | 6x_s  6x_s  6x_s  6x_s  6x_s | X  X  X  X  X | H2  H2  H2  H2  H2 | KY034455  KY034456  KY034457  KY034458  KY034459 | N7  N7  N7  N7  N7 | KY009257  KY009258  KY009259  KY009260  KY009261 | FR-0121747  -  -  -  - |
|  | CRA1 | CB_PAL6 | | FRA, Bas-Rhin, Gimbelhof; KUN | 4x_s | X | H2 | KY034569 | N7 | KY009379 | FR-0121761 |
|  | CKF1  CKF2  CKF3  CKF4 | CB_PAL8  CB_PAL8  CB_PAL8  CB_PAL8 | | FRA, Bas-Rhin, Wengelsbach; KUN  FRA, Bas-Rhin, Wengelsbach; KUN  FRA, Bas-Rhin, Wengelsbach; KUN  FRA, Bas-Rhin, Wengelsbach; KUN | 4x_s  4x_s  4x_s  4x_s | X  X  X  X | H2  H2  H2  H2 | KY034527  KY034528  KY034529  KY034530 | N7  N7  N7  N7 | KY009332  KY009333  KY009334  KY009335 | FR-0121754  -  -  - |
|  | CNO1  CNO2  CNO3  CNO4  CNO5  CNO6  CNO7  CNO8  CNO9  CNO10  CNO11  CNO12 | CB_PAL9 CB_PAL9  CB_PAL9  CB_PAL9  CB_PAL9  CB_PAL9  CB_PAL9  CB_PAL9  CB_PAL9  CB_PAL9  CB_PAL9  CB_PAL9 | | DEU, Rhineland-Palatinate, Nothweiler; KUN  DEU, Rhineland-Palatinate, Nothweiler; KUN  DEU, Rhineland-Palatinate, Nothweiler; KUN  DEU, Rhineland-Palatinate, Nothweiler; KUN  DEU, Rhineland-Palatinate, Nothweiler; KUN  DEU, Rhineland-Palatinate, Nothweiler; KUN  DEU, Rhineland-Palatinate, Nothweiler; KUN  DEU, Rhineland-Palatinate, Nothweiler; KUN  DEU, Rhineland-Palatinate, Nothweiler; KUN  DEU, Rhineland-Palatinate, Nothweiler; KUN  DEU, Rhineland-Palatinate, Nothweiler; KUN  DEU, Rhineland-Palatinate, Nothweiler; KUN | 4x_s  4x_s  4x_s  4x_s  4x_s  4x_s  4x_s  4x_s  4x_s  4x_s  4x_s  4x_s | X  X  X  X  X  X  X  X  X  X  X  X | H2  H2  H2  H2  H2  H2  H2  H2  H2  H6  H2  H2 | KY034545  KY034546  KY034547  KY034548  KY034549  KY034550  KY034551  KY034552  KY034553  KY034554  KY034555  KY034556 | N7  N7  N7  N7  N7  N7  N7  N7  N7  N7  N4  N7 | KY009350  KY009351  KY009352  KY009353  KY009354  KY009355  KY009356  KY009357  KY009358  KY009359  KY009360  KY009361 | FR-0121760  -  -  -  -  -  -  -  -  -  -  - |
|  | CAMP01 | CB_PAL12 | | DEU, Rhineland-Palatinate, Blankenborn; TG | 6x_cc | - | - | - | - | - | - |
| ***Campanula baumgartenii* x *rotundifolia*** | | | | | | | | | | | |
|  | COW1 | X_TAU4 | | DEU, Hesse, Oberreifenberg; KUN | 5x_s | X | H2 | KY034563 | N7 | KY009368 | FR-0121730 |
|  | CHK2 | X_TAU7 | | DEU, Hesse, Oberreifenberg; KUN | 5x_s | X | H5 | KY034497 | N7 | KY009298 | - |
|  | CSK2  CSK3  CSK8 | X_TAU12  X_TAU12  X_TAU12 | | DEU, Hesse, Oberreifenberg; KUN  DEU, Hesse, Oberreifenberg; KUN  DEU, Hesse, Oberreifenberg; KUN | 5x_s_cc  5x_s  5x_s | X  X  X | H8  H2  H2 | KY034604  KY034605  KY034610 | N7  N7  N7 | KY009414  KY009415  KY009420 | -  -  - |
|  | CSW1  CSW3 | X_TAU15  X_TAU15 | | DEU, Hesse, Oberreifenberg; KUN  DEU, Hesse, Oberreifenberg; KUN | 5x_s  5x_s | X  X | H2  H7 | KY034618  KY034620 | N7  N7 | KY009427  KY009429 | FR-0121738  - |
|  | CHW5  CHW7 | X_TAU19  X_TAU19 | | DEU, Hesse, Oberreifenberg; KUN  DEU, Hesse, Oberreifenberg; KUN | 5x_s  5x_s | X  X | H2  H2 | KY034523  KY034525 | N7  N7 | KY009324  KY009326 | -  - |
|  | CGF5 | X_TAU21 | | DEU, Hesse, Großer Feldberg; KUN | 5x_s | X | H2 | KY034470 | N7 | KY009272 | - |
| ***Campanula rotundifolia*** | | | | | | | | | | | |
|  | CSA1  CSA2  CSA3  CSA4 | CR_TAU1  CR_TAU1  CR_TAU1  CR_TAU1 | DEU, Hesse, Hegewiese; KUN  DEU, Hesse, Hegewiese; KUN  DEU, Hesse, Hegewiese; KUN  DEU, Hesse, Hegewiese; KUN | | 4x_s  4x_s  4x_s  4x_s | X  X  X  X | H7  H7  H7  H7 | KY034578  KY034579  KY034580  KY034581 | N7  N7  N7  N7 | KY009388  KY009389  KY009390  KY009391 | FR-0121733  -  -  - |
|  | CBR1 | CR_TAU8 | DEU, Hesse, Großer Feldberg; KUN | | 4x_s | X | H7 | KY034460 | N7 | KY009262 | FR-0121737 |
|  | CSC1  CSC2  CSC3  CSC4  CSC5 | CR_TAU10  CR_TAU10  CR_TAU10  CR_TAU10  CR_TAU10 | DEU, Hesse, Niederreifenberg; KUN  DEU, Hesse, Niederreifenberg; KUN  DEU, Hesse, Niederreifenberg; KUN  DEU, Hesse, Niederreifenberg; KUN  DEU, Hesse, Niederreifenberg; KUN | | 4x_s  4x_s  4x_s  4x_s  4x_s | X  X  X  X  X | H7  H7  H7  H7  H7 | KY034589  KY034590  KY034591  KY034592  KY034593 | N7  N7  N7  N7  N7 | KY009399  KY009400  KY009401  KY009402  KY009403 | FR-0121735  -  -  -  - |
|  | CSP1  CSP2  CSP3 | CR_TAU14  CR_TAU14  CR_TAU14 | DEU, Hesse, Oberreifenberg; KUN  DEU, Hesse, Oberreifenberg; KUN  DEU, Hesse, Oberreifenberg; KUN | | 4x_s  4x_s  4x_s | X  X  X | H8  H7  H7 | KY034615  KY034616  KY034617 | N7  N7  N7 | KY009424  KY009425  KY009426 | FR-0121739  -  - |
|  | CSW4 | CR_TAU15 | DEU, Hesse, Oberreifenberg; KUN | | 4x_s | X | H7 | KY034621 | N7 | KY009430 | - |
|  | CSH8 | CR_TAU17 | DEU, Hesse, Oberreifenberg; KUN | | 4x_s | X | H7 | KY034601 | N7 | KY009411 | FR-0121744 |
|  | CHW1  CHW2  CHW3 | CR_TAU18  CR_TAU18  CR_TAU18 | DEU, Hesse, Oberreifenberg; KUN  DEU, Hesse, Oberreifenberg; KUN  DEU, Hesse, Oberreifenberg; KUN | | 4x_s  4x_s  4x_s | X  X  X | H8  H8  H7 | KY034519  KY034520  KY034521 | N7  N7  N7 | KY009320  KY009321  KY009322 | FR-0121743  -  - |
|  | CWJ1  CWJ2  CWJ3  CWJ4  CWJ5 | CR_TAU20  CR_TAU20  CR_TAU20  CR_TAU20  CR_TAU20 | DEU, Hesse, Niedernhausen; KUN  DEU, Hesse, Niedernhausen; KUN  DEU, Hesse, Niedernhausen; KUN  DEU, Hesse, Niedernhausen; KUN  DEU, Hesse, Niedernhausen; KUN | | 4x_s  4x_s  4x_s  4x_s  4x_s | X  X  X  X  X | H7  H7  H7  H7  H7 | KY034632  KY034633  KY034634  KY034635  KY034636 | N7  N7  N7  N7  N7 | KY009440  KY009441  KY009442  KY009443  KY009444 | FR-0121762  -  -  -  - |
|  | CGF1  CGF3  CGF4 | CR_TAU21  CR_TAU21  CR_TAU21 | DEU, Hesse, Großer Feldberg; KUN  DEU, Hesse, Großer Feldberg; KUN  DEU, Hesse, Großer Feldberg; KUN | | 4x_s  4x_s  4x_s | X  X  X | H9  H9  H9 | KY034466  KY034468  KY034469 | N7  N7  N7 | KY009268  KY009270  KY009271 | FR-0121772  -  - |
|  | CGF9  CGF10  CGF11 | CR_TAU23  CR_TAU23  CR_TAU23 | DEU, Hesse, Großer Feldberg; KUN  DEU, Hesse, Großer Feldberg; KUN  DEU, Hesse, Großer Feldberg; KUN | | 4x_s  4x_s  4x_s | X  X  X | H10  H7  H7 | KY034474  KY034475  KY034476 | N7  N7  N7 | KY009275  KY009276  KY009277 | FR-0121770  -  - |
|  | CPW1  CPW2  CPW3 | CR_PAL2  CR_PAL2  CR_PAL2 | DEU, Rhineland-Palatinate, Hauenstein; KUN  DEU, Rhineland-Palatinate, Hauenstein; KUN  DEU, Rhineland-Palatinate, Hauenstein; KUN | | 4x_s  4x_s  4x_s | X  X  X | H7  H7  H7 | KY034566  KY034567  KY034568 | N1  N2  N1 | KY009371  KY009372  KY009373 | FR-0121750  -  - |
|  | CHS1  CHS2 | CR_PAL4  CR_PAL4 | DEU, Rhineland-Palatinate, Hochspeyer; KUN  DEU, Rhineland-Palatinate, Hochspeyer; KUN | | 4x_s  4x_s | X  X | H8  H8 | KY034503  KY034504 | N7  N7 | KY009304  KY009305 | FR-0121746  - |
|  | CVW1  CVW2  CVW3 | CR_PAL5  CR_PAL5  CR_PAL5 | DEU, Rhineland-Palatinate, Vorderweidenthal; KUN  DEU, Rhineland-Palatinate, Vorderweidenthal; KUN  DEU, Rhineland-Palatinate, Vorderweidenthal; KUN | | 4x_s  4x_s  4x_s | X  X  X | H7  H7  H7 | KY034622  KY034623  KY034624 | N7  N5  N7 | KY009431  KY009432  KY009433 | FR-0121757  -  - |
|  | CPF1  CPF2 | CR_PAL6  CR_PAL6 | FRA, Bas-Rhin, Gimbelhof; KUN  FRA, Bas-Rhin, Gimbelhof; KUN | | 2x_s  2x_s | X  X | H10  H10 | KY034564  KY034565 | N7  N7 | KY009369  KY009370 | FR-0121756  - |
|  | CHB1  CHB2 | CR_PAL7  CR_PAL7 | DEU, Rhineland-Palatinate, Hirschthal; KUN  DEU, Rhineland-Palatinate, Hirschthal; KUN | | 4x_s  4x_s | X  X | H7  H7 | KY034490  KY034491 | N7  N7 | KY009291  KY009292 | FR-0121755  - |
|  | CKF5 | CR_PAL8 | FRA, Bas-Rhin, Wengelsbach; KUN | | 4x_s | X | H7 | KY034531 | N7 | KY009336 | - |
|  | CHI1 | CR_PAL10 | DEU, Rhineland-Palatinate, Hinterweidenthal; KUN | | 4x_s | X | H7 | KY034495 | N6 | KY009296 | FR-0121759 |
|  | CNW1  CNW2  CNW3  CNW4  CNW5  CNW6 | CR_PAL11  CR_PAL11  CR_PAL11  CR_PAL11  CR_PAL11  CR_PAL11 | DEU, Rhineland-Palatinate, Neustadt/Weinstraße; KUN  DEU, Rhineland-Palatinate, Neustadt/Weinstraße; KUN  DEU, Rhineland-Palatinate, Neustadt/Weinstraße; KUN  DEU, Rhineland-Palatinate, Neustadt/Weinstraße; KUN  DEU, Rhineland-Palatinate, Neustadt/Weinstraße; KUN  DEU, Rhineland-Palatinate, Neustadt/Weinstraße; KUN | | 4x_s  4x_s  4x_s  4x_s  4x_s  4x_s | X  X  X  X  X  X | H7  H7  H7  H7  H7  H8 | KY034557  KY034558  KY034559  KY034560  KY034561  KY034562 | N7  N7  N7  N7  N5  N7 | KY009362  KY009363  KY009364  KY009365  KY009366  KY009367 | FR-0121758  -  -  -  -  - |
|  | CLW1  CLW2  CLW3 | CR_URR1  CR_URR1  CR_URR1 | DEU, Hesse, Langen; KUN  DEU, Hesse, Langen; KUN  DEU, Hesse, Langen; KUN | | 2x_s  2x_s  2x_s | X  X  X | H10  H10  H10 | KY034542  KY034543  KY034544 | N7  N7  N7 | KY009347  KY009348  KY009349 | FR-0121752  -  - |
|  | CHE1  CHE2  CHE3 | CR_URR2  CR_URR2  CR_URR2 | DEU, Hesse, Langen; KUN  DEU, Hesse, Langen; KUN  DEU, Hesse, Langen; KUN | | 2x_s  2x_s  2x_s | X  X  X | H10  H10  H10 | KY034492  KY034493  KY034494 | N3  N7  N7 | KY009293  KY009294  KY009295 | FR-0121751  -  - |
| ***Campanula scheuchzeri*** | | | | | | | | | | | |
|  | CHA1  CHA2  CHA5 | CS_ALP1  CS_ALP1  CS_ALP1 | | AUT, Tirol, Hahntennjoch; KUN  AUT, Tirol, Hahntennjoch; KUN  AUT, Tirol, Hahntennjoch; KUN | 4x  4x  4x | X  X  X | H1  H1  H1 | KY034485  KY034486  KY034489 | N7  N7  N7 | KY009286  KY009287  KY009290 | FR-0121769  -  - |
|  | CRM1  CRM2  CRM3  CRM4  CRM5  CRM6  CRM7  CRM8 | CS_ALP2  CS_ALP2  CS_ALP2  CS_ALP2  CS_ALP2  CS_ALP2  CS_ALP2  CS_ALP2 | | AUT, Tirol, Obergurgl; KUN  AUT, Tirol, Obergurgl; KUN  AUT, Tirol, Obergurgl; KUN  AUT, Tirol, Obergurgl; KUN  AUT, Tirol, Obergurgl; KUN  AUT, Tirol, Obergurgl; KUN  AUT, Tirol, Obergurgl; KUN  AUT, Tirol, Obergurgl; KUN | 4x  4x  4x  4x  4x  4x  4x  4x | X  X  X  X  X  X  X  X | H1  H1  H1  H1  H1  H1  H1  H1 | KY034570  KY034571  KY034572  KY034573  KY034574  KY034575  KY034576  KY034577 | N7  N7  N7  N7  N7  N7  N7  N7 | KY009380  KY009381  KY009382  KY009383  KY009384  KY009385  KY009386  KY009387 | FR-0121767  -  -  -  -  -  -  - |
|  | CGT6  CGT7  CGT8 | CS_ALP3  CS_ALP3  CS_ALP3 | | AUT, Tirol, Obergurgl; KUN  AUT, Tirol, Obergurgl; KUN  AUT, Tirol, Obergurgl; KUN | 4x  4x  4x | X  X  X | H1  H1  H1 | KY034482  KY034483  KY034484 | N7  N7  N7 | KY009283  KY009284  KY009285 | FR-0121765  -  - |
| ***Campanula cochleariifolia*** | | | | | | | | | | | |
|  | CHA3  CHA4 | CC_ALP1  CC_ALP1 | | AUT, Tirol, Hahntennjoch; KUN  AUT, Tirol, Hahntennjoch; KUN | 2x  2x | X  X | H12  H13 | KY034487  KY034488 | N8  N8 | KY009288  KY009289 | FR-0121768  - |
|  | CGT1  CGT2  CGT3  CGT4  CGT5 | CC_ALP3  CC_ALP3  CC_ALP3  CC_ALP3  CC_ALP3 | | AUT, Tirol, Obergurgl; KUN  AUT, Tirol, Obergurgl; KUN  AUT, Tirol, Obergurgl; KUN  AUT, Tirol, Obergurgl; KUN  AUT, Tirol, Obergurgl; KUN | 2x  2x  2x  2x  2x | X  X  X  X  X | H11  H11  H11  H11  H11 | KY034477  KY034478  KY034479  KY034480  KY034481 | N9  N10  N10  N10  N10 | KY009278  KY009279  KY009280  KY009281  KY009282 | FR-0121766  -  -  -  - |

**Table S2** Spearman‘s correlation coefficients (rho) between 20 characters measured and scored for studied *Campanula* accessions. Bold font highlights the absolute values greater than 0.95. For character coding see Table S2.

|  | **1** | **2** | **3** | **4** | **5** | **6** | **7** | **8** | **9** | **10** | **11** | **12** | **13** | **14** | **15** | **16** | **17** | **18** | **19** | **20** |
| --- | --- | --- | --- | --- | --- | --- | --- | --- | --- | --- | --- | --- | --- | --- | --- | --- | --- | --- | --- | --- |
| **1** |  | | | | | | | | | | | | | | | | | | | |
| **2** | 0.271 |  |  |  |  |  |  |  |  |  |  |  |  |  |  |  |  |  |  |  |
| **3** | -0.375 | -0.469 |  |  |  |  |  |  |  |  |  |  |  |  |  |  |  |  |  |  |
| **4** | 0.452 | 0.458 | -0.189 |  |  |  |  |  |  |  |  |  |  |  |  |  |  |  |  |  |
| **5** | 0.237 | -0.053 | 0.153 | 0.222 |  |  |  |  |  |  |  |  |  |  |  |  |  |  |  |  |
| **6** | -0.203 | -0.074 | 0.254 | 0.045 | 0.052 |  |  |  |  |  |  |  |  |  |  |  |  |  |  |  |
| **7** | 0.182 | -0.009 | -0.269 | -0.201 | -0.146 | -0.725 |  |  |  |  |  |  |  |  |  |  |  |  |  |  |
| **8** | -0.235 | -0.228 | 0.297 | -0.303 | 0.081 | 0.051 | -0.081 |  |  |  |  |  |  |  |  |  |  |  |  |  |
| **9** | -0.007 | -0.138 | -0.114 | -0.139 | -0.311 | -0.074 | 0.537 | -0.180 |  |  |  |  |  |  |  |  |  |  |  |  |
| **10** | -0.160 | -0.199 | -0.077 | -0.324 | -0.357 | 0.154 | 0.317 | -0.087 | 0.862 |  |  |  |  |  |  |  |  |  |  |  |
| **11** | -0.195 | -0.240 | 0.227 | -0.329 | 0.041 | -0.081 | 0.057 | **0.951** | -0.087 | -0.018 |  |  |  |  |  |  |  |  |  |  |
| **12** | 0.305 | 0.467 | -0.374 | 0.208 | 0.028 | -0.136 | 0.144 | -0.424 | -0.039 | 0.004 | -0.417 |  |  |  |  |  |  |  |  |  |
| **13** | -0.239 | -0.155 | 0.143 | -0.104 | -0.014 | 0.166 | -0.330 | 0.549 | -0.164 | 0.011 | 0.645 | -0.416 |  |  |  |  |  |  |  |  |
| **14** | -0.260 | -0.360 | 0.282 | -0.631 | 0.196 | 0.090 | 0.125 | 0.106 | 0.115 | 0.161 | 0.036 | -0.126 | -0.164 |  |  |  |  |  |  |  |
| **15** | -0.001 | -0.212 | 0.176 | 0.042 | 0.654 | 0.040 | 0.016 | 0.147 | -0.039 | -0.059 | 0.179 | -0.120 | 0.189 | 0.148 |  |  |  |  |  |  |
| **16** | 0.216 | -0.259 | 0.217 | 0.091 | 0.760 | -0.052 | 0.113 | -0.022 | 0.031 | -0.037 | -0.007 | -0.074 | -0.025 | 0.247 | 0.695 |  |  |  |  |  |
| **17** | 0.247 | 0.365 | -0.334 | 0.311 | 0.018 | 0.033 | -0.137 | -0.399 | -0.140 | -0.159 | -0.459 | 0.337 | -0.447 | -0.044 | -0.342 | -0.162 |  |  |  |  |
| **18** | 0.377 | 0.552 | -0.446 | 0.627 | 0.019 | -0.131 | -0.145 | -0.356 | -0.248 | -0.307 | -0.317 | 0.346 | -0.142 | -0.580 | -0.239 | -0.172 | 0.650 |  |  |  |
| **19** | -0.063 | -0.531 | 0.430 | -0.238 | 0.207 | 0.019 | 0.147 | 0.069 | 0.195 | 0.246 | 0.097 | -0.212 | 0.008 | 0.307 | 0.104 | 0.440 | -0.232 | -0.362 |  |  |
| **20** | 0.263 | -0.152 | 0.053 | 0.077 | 0.528 | -0.115 | 0.081 | 0.184 | 0.038 | 0.025 | 0.224 | -0.167 | 0.175 | 0.109 | 0.350 | 0.580 | 0.065 | 0.014 | 0.574 |  |
